# Clinical feasibility of fast adaptive four-dimensional cone-beam computed tomography for lung cancer radiotherapy

**DOI:** 10.1016/j.phro.2026.101007

**Published:** 2026-05-26

**Authors:** Sadia Sana, Shalini K. Vinod, Paul Keall, Vicky Chin, Nabeeha Chowdhury, Jan-Jakob Sonke, Christine Tawfik, Isabella Franji, Rebecca Bartlett, Vinh Luong-Poole, Owen Dillon, Ricky T. O’Brien

**Affiliations:** a School of Health and Biomedical Sciences, RMIT University, Melbourne, Australia; b Liverpool & Macarthur Cancer Therapy Centers, Liverpool Hospital, Liverpool, New South Wales, Australia; c South Western Sydney Clinical School, The University of New South Wales & Ingham Institute for Applied Medical Research, Liverpool, New South Wales, Australia; d Image X Institute, School of Health Sciences, University of Sydney, NSW, Australia; e Department of Radiation Oncology, Netherlands Cancer Institute, Amsterdam 1066 CX, The Netherlands

**Keywords:** Adaptive 4DCBCT, Lung cancer radiotherapy, Motion-compensated reconstruction, Dose reduction, Tumor visibility, And Image Quality

## Abstract

**Background and purpose:**

Adaptive Four-Dimensional Cone-Beam Computed Tomography (4DCBCT) can reduce scan time and imaging dose in radiotherapy. This is achieved by modulating the projection acquisition rate and gantry rotation speed in response to real-time changes in patient breathing, together with motion-compensated image reconstruction. This study aimed to evaluate the clinical image quality of a fast adaptive 4DCBCT acquisition compared with conventional 4DCBCT in the treatment of lung cancer.

**Materials and methods:**

Image datasets from the Adaptive 4DCBCT (ADAPT) clinical trial (ACTRN12618001440213), which included 30 patients treated for lung cancer, were analyzed. Two scan types were assessed: fast adaptive 4DCBCT (200 projections acquired over 20 breathing cycles, approximately 60–80s) and conventional 4DCBCT (1320 projections acquired over approximately 80 breathing cycles, 4 min). Two radiation oncologists and four radiation therapists, blinded to the image acquisition technique, independently rated the clinical utility of each scan using a two-question survey. Tumor visibility was rated on a three-point scale, and overall image quality was rated on a ten-point scale. A paired *t*-test was used to compare scores across acquisition techniques.

**Results:**

Fast adaptive 4DCBCT showed a mean tumor visibility score of 2.3 ± 0.8, compared to conventional 4DCBCT (2.4 ± 0.7). For general image quality, fast adaptive 4DCBCT achieved a mean score of 4.1 ± 1.3, compared to conventional 4DCBCT, which had a mean score of 4.3 ± 1.2. There were no statistically significant differences in tumor visibility or image quality scores between fast adaptive 4DCBCT and conventional 4DCBCT scans.

**Conclusion:**

Fast adaptive 4DCBCT achieved similar image quality scores to conventional 4DCBCT while requiring only 15% of the imaging dose and 25% of the scan time. This study confirms the clinical feasibility of adaptive scanning protocols for use in radiation therapy for lung cancer.

## Highlights

• Adaptive cone beam CT enables one-minute lung imaging.
• Acquisition shortened from 240 s to approximately 70 s.
• Imaging dose is approximately 85 percent lower than the standard protocol.
• Maintains clinically acceptable tumor visibility for treatment guidance.

## Graphical abstract

**Figure:**
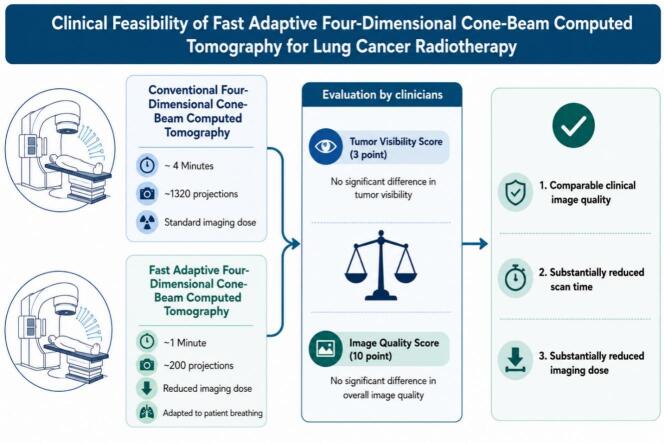

## 1 Introduction

Effective management of lung cancer through radiation therapy is often challenged by tumor motion caused by respiration, which can result in reduced targeting accuracy and compromised treatment outcomes [1]. Four-dimensional cone-beam computed tomography (4DCBCT) has been widely adopted to address this issue by providing volumetric imaging synchronized with the patient’s respiratory cycle. The European Society for Radiotherapy and Oncology (ESTRO) Advisory Committee on Radiation Oncology Practice (ACROP) consensus guideline recommends advanced imaging modalities, such as 4DCBCT, for accurate tumor localization and motion assessment in stereotactic ablative radiotherapy (SABR) for lung cancer [2]. The introduction of 4DCBCT has enabled improved visualization of tumor motion, supporting more accurate image guidance across a range of thoracic radiotherapy settings [3]. However, conventional 4DCBCT techniques are associated with significant limitations, including prolonged acquisition times [4], and increased radiation doses [5], [6], [7], which limit clinical adoption [8], [9].

An adaptive 4DCBCT protocol has been proposed to overcome these drawbacks. These protocols aim to reduce imaging dose and acquisition time while maintaining diagnostic image quality suitable for clinical use [10], [11]. This protocol was developed within the Adaptive CT acquisition for personalized thoracic imaging (ADAPT) clinical trial, a pilot study designed to investigate respiratory motion-guided 4DCBCT for lung cancer radiotherapy. Within this framework, the ADAPT protocols adapt gantry rotation speed and projection acquisition in response to real-time changes in the patient’s breathing rate [12] and leverage motion-compensated reconstruction techniques [13]. These reconstruction approaches have demonstrated the ability to capture tumor motion from substantially fewer projections compared with conventional reconstruction algorithms [11], [14]. Despite these advancements and quantitative studies on adaptive 4DCBCT, the clinical feasibility of the approach still needs to be assessed by experienced clinicians.

The aim of this study was to perform a qualitative evaluation of adaptive 4DCBCT imaging to ensure consistency with previously reported quantitative findings. By integrating visual analyses, this study investigates the feasibility of replacing conventional 4DCBCT with faster adaptive protocols without compromising image quality. The goal was to support evidence-based improvements in imaging efficiency while maintaining clinical standards in lung cancer radiotherapy.

## 2 Materials and methods

### 2.1 Study design and patient cohort

This study reports on the primary hypothesis of the ADAPT clinical trial (ACTRN12618001440213, ethics approval 2019/ETH09968), in which all participants provided written informed consent before enrolment. The consent process explicitly included information regarding the additional imaging procedures, time for the scans, and associated imaging dose. The trial hypothesized that fast adaptive 4DCBCT (60–80s, 200 projections) was able to produce equivalent image quality to conventional 4DCBCT (4 min, 1320 projections), with significant reductions in scan time and dose.

Imaging data were acquired from 30 patients treated at Liverpool Hospital, Australia. The cohort included patients treated with stereotactic ablative radiotherapy and conventionally fractionated thoracic radiotherapy. In accordance with institutional practice, stereotactic patients underwent daily 4DCBCT, while conventionally fractionated patients underwent 4DCBCT on the first fraction and weekly thereafter. This provided a clinically representative cohort to evaluate fast adaptive 4DCBCT under routine conditions.

The reference planning CT dataset used for contouring was derived from the patient's 4DCT simulation scan, performed as part of routine radiotherapy planning. At each fraction, patients underwent both a fast adaptive and a conventional 4DCBCT scan. All imaging was performed during routine treatment sessions to avoid additional patient visits. In this study, each scan pair was analyzed independently. A detailed description of the imaging protocols is provided in Section 2.2.

Due to patient and technical factors, not all fractions included both scan types. A total of 60 fractions were planned (30 patients × 2), with paired adaptive and conventional 4DCBCT acquired in 54 fractions. In the remaining six fractions, one scan was not acquired due to patient discomfort or technical interruptions during acquisition. Only fractions with paired scans (n = 54) were included in the analysis.

Table 1 presents the demographic and clinical characteristics of the 30 patients included. The cohort comprised 15 males and 15 females, with ages ranging from 49 to 85 years (mean 69.3). Body weight varied considerably, with a mean of 75.7 kg (range 41.0 to 129.0 kg). In terms of tumor staging, most patients had T1 tumors (n = 9), followed by T2 (n = 5), T4 (n = 4), T3 (n = 2), and Tx (n = 2). Overall, 10 patients were Stage I, 14 Stage II/III, and 6 Stage IV or recurrent disease treated with moderate to high radiotherapy doses.

**Table 1:** Demographic and clinical characteristics of the patients enrolled in the study. TNM = Tumor–Node–Metastasis classification system; T = primary tumor extent, N = regional lymph node involvement, M = distant metastasis.

Age (Years)	Sex	Weight (kg)	TNM Stage	Cancer Stage
71	Male	94.4	T2N2M0	Stage III
65	Male	60.4	T1N3M0	Stage III
59	Female	63.8	T3N2M0	Stage III
49	Female	55.0	T4N2M0	Stage III
82	Female	58.7	T1N0M0	Stage I
51	Male	82.8	TXNXM1	Stage IV
78	Female	55.9	T1N0M0	Stage I
58	Female	95.1	T1N2M1	Stage IV
85	Male	79.4	T4N1M0	Stage III
76	Male	86.5	T3N3M0	Stage III
52	Female	70.1	T2N1M1	Stage IV
82	Male	73.0	T4N1M0	Stage III
74	Female	52.2	T1N0M0	Stage I
78	Male	93.0	T1N0M0	Stage I
78	Male	70.9	T1N0M0	Stage I
71	Male	75.5	T2N0MX	Stage I
77	Female	59.9	T1N0M0	Stage I
78	Male	69.0	T1N0M0	Stage I
79	Female	129.0	TXNXMX	Recurrence
63	Male	81.4	T4N2M0	Stage III
67	Male	74.6	T2N1M0	Stage II
53	Female	123.4	T2N2M0	Stage III
63	Female	41.0	T2N3M1	Stage IV
72	Male	62.5	T0N3MX	Stage III
77	Female	55.0	T1N0M0	Stage I
81	Female	84.0	T1N0M0	Stage I
69	Female	44.6	T1N2M0	Stage III
74	Female	75.0	T4N0M1	Stage IV
59	Male	81.0	T2N1M0	Stage II
70	Male	83.8	TXN2M0	Stage III

### 2.2 Imaging protocols

**Fig. 1:**
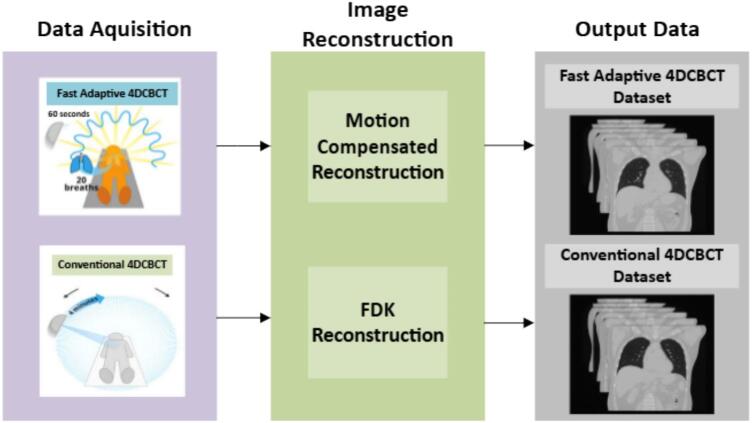
Overview of 4DCBCT datasets: fast adaptive and conventional acquisition protocols. FDK, Feldkamp–Davis–Kress reconstruction algorithm.

The adaptive 4DCBCT protocol has been previously described in detail [5], [11], [12], [14], [15], [16], [17]. Briefly, the acquisition was synchronized with respiratory motion using a depth-sensing camera. Gantry rotation speed was dynamically adjusted according to respiratory state, and motion-compensated reconstruction was applied as previously reported.

(Fig. 1) shows a schematic of how the 4DCBCT datasets were acquired and reconstructed for the ADAPT clinical trial.

The fast adaptive protocol acquired 200 projections over a 200° gantry rotation, with projections distributed across 10 respiratory phases and reconstructed using motion-compensated image reconstruction (MC-MKB) [14]. Scan duration was patient-specific and, in general, approximately 60–80s, depending on breathing rate [14], [16]. The choice of 200 projections was informed by prior simulation studies [14], [17], which showed that adaptive acquisition with MC-MKB can produce comparable image quality to conventional 4DCBCT.

Conventional 4DCBCT was acquired using standard protocols over a 200° gantry rotation with 1320 projections during a 4 min acquisition. Projections were retrospectively sorted into 10 respiratory phases using the Amsterdam Shroud method [10], [18] and reconstructed with the Feldkamp–Davis–Kress algorithm [19].

The reduction in projection number relative to conventional acquisition was calculated to estimate proportional differences in imaging exposure. The fast adaptive protocol acquired 200 projections compared with approximately 1320 for conventional 4DCBCT. Exposure parameters, including tube voltage and tube current, were consistent between protocols in accordance with institutional standards. No protocol-specific adjustment based on patient body size was applied beyond standard clinical practice.

### 2.3 Image suitability assessment procedure

Image quality was evaluated using a blinded assessment by experienced clinicians. To achieve a high level of reliability and validity in the assessment process, two radiation oncologists and four radiation therapists were recruited to conduct the survey. All of the assessors had at least five years of clinical experience in lung cancer radiotherapy. Assessors were blinded to the imaging acquisition techniques and evaluated image suitability using a two-question survey assessing tumor visibility and image quality. The tumor visibility assessment was adapted from a study by Sweeney RA et al. (2012) [20] (see Table 2).

**Table 2:** Assessment criteria for tumor visibility and image quality.

Assessment	Score	Definition
Tumor Visibility	1	Precise visual localization of the target is difficult or not possible for visual verification of image guidance.
	2	A visible tumor with difficulties in manual verification of image guidance results.
	3	A clearly visible tumor without any difficulties in manual verification of image guidance results.
Image Quality	1–10	Rated on a 10-point scale: 1 = worst image quality encountered in clinical practice; 5 = typical clinical image quality; 10 = exceptionally high image quality.

### 2.4 Survey protocol for image quality evaluation

To eliminate bias, all assessors were masked to the image acquisition methods. Each assessor received anonymized image datasets, including all scan types from patients 1–30, presented in randomized order. Datasets were assigned randomized codes and shuffled using a computerized script to ensure masking and prevent sequential pattern recognition.

All datasets were stripped of metadata and renamed with randomized alphanumeric codes before upload to a Google Form survey. Assessors accessed images via embedded links, ensuring evaluations were based solely on visual and clinical image quality without identifiable or sequential cues. Static image datasets were extracted from each respiratory phase and included axial, sagittal, and coronal planes centered on the internal target volume (ITV). For each phase, representative slices through the ITV centroid were selected to ensure consistent anatomical comparison across scan types. Assessors were presented with multi-planar reconstructions across all ten respiratory phases for each dataset. In addition, animated Graphics Interchange Format (GIF) images of each plane through the ITV center, displaying sequential respiratory phases, were provided to illustrate tumor motion. The ITV and spinal canal contours were transferred from the reference four-dimensional computed tomography planning dataset to the corresponding 4DCBCT datasets using rigid image registration in MiM Maestro (MiM Software Inc., Cleveland, OH, USA), a commercially available platform used in radiotherapy planning. Following registration, structure sets were propagated to the 4DCBCT and adaptive 4DCBCT images for visual overlay.

These overlays were provided solely to aid visual localization and were identical across imaging modalities. For a comprehensive review, anonymized CT and 4DCBCT image pairs were also available within the MiM platform for optional detailed assessment.

Data was exported from Google Forms to Microsoft Excel and screened for missing entries, duplicates, and inconsistencies. Incomplete responses were excluded, and duplicates, identified by assessor ID and timestamp, were removed, retaining the first valid entry. Outliers, defined as values exceeding two standard deviations from the mean, were visually inspected and cross-checked against the original records. If these deviations resulted from data entry variations, they were excluded; otherwise, they were retained as valid extreme observations. The cleaned dataset was structured in Microsoft Excel (Microsoft Corp., Redmond, WA, USA) and subsequently analysed using Python version 3.10 (Python Software Foundation, Wilmington, DE, USA).

**Fig. 2:**
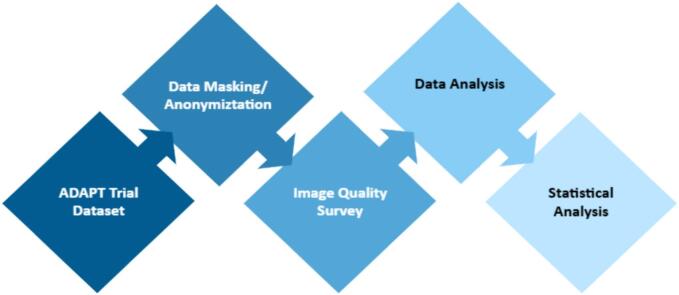
Analysis workflow including image quality assessment, data analysis, and statistical analysis (*t*-test, p-value, and hypothesis evaluation).

A visual representation of the evaluation and analysis workflow, illustrating the systematic approach, is provided in (Fig. 2).

### 2.5 Statistical analysis

Summary statistics were calculated across each scan type. To compare the clinical image quality between fast adaptive 4DCBCT and conventional 4DCBCT, a pairwise analysis was performed. A paired samples *t*-test to test the null hypothesis that no difference exists between the allocated scores. Statistical significance was defined as p < 0.05. Inter-observer agreement for tumor visibility was assessed using Fleiss’ kappa, given the categorical three-point rating scale. Reliability of image quality scoring was evaluated using the intraclass correlation coefficient (two-way random effects model, absolute agreement).

## 3 Results

**Fig. 3:**
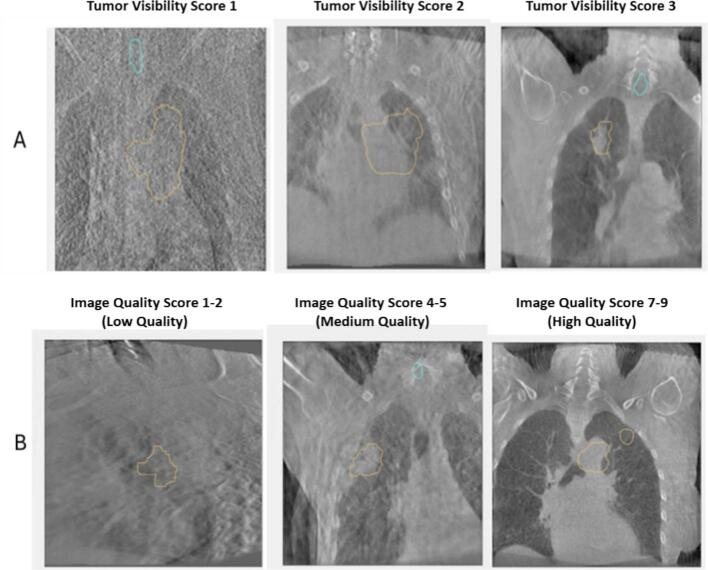
Representative examples of (A) tumor visualization scores and (B) image quality scores as determined by the assessors. Note: The yellow contour indicates the tumor (ITV), and the blue contour marks the spine. (For interpretation of the references to colour in this figure legend, the reader is referred to the web version of this article.)

**Fig. 4:**
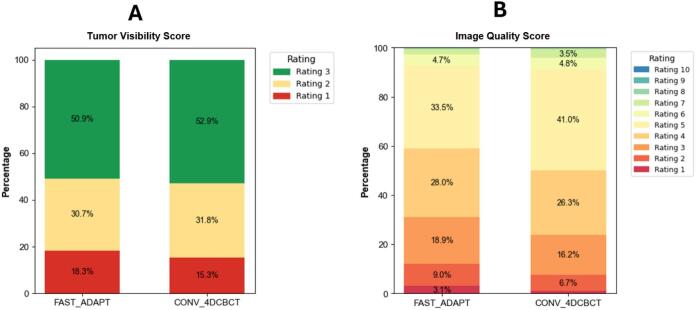
Rating distributions of imaging techniques: A: Tumor visibility score, B: Image quality score.

Representative examples of tumor visibility categories and image quality scores are shown in (Fig. 3), illustrating the range of image appearances corresponding to the predefined scoring criteria.

(Fig. 4) shows the distribution of tumor visibility and image quality scores across imaging protocols. (Fig. 4A) corresponds to tumor visibility (scale 1–3) and (Fig. 4B) to image quality (scale 1–10).

For tumor visibility, the fast adaptive protocol yielded scores of 1 (18.3%), 2 (30.7%), and 3 (50.9%), compared with 1 (15.3%), 2 (31.8%), and 3 (52.9%) for conventional 4DCBCT. For image quality, the fast adaptive protocol most frequently received scores of 5 (33.5%), 4 (28.0%), and 3 (18.9%), while conventional 4DCBCT received scores of 5 (41.0%), 4 (26.3%), and 3 (16.2%).

The tumor visibility score for fast adaptive 4DCBCT was 2.3 ± 0.8, and for conventional 4DCBCT was 2.4 ± 0.7, with the standard deviation relatively similar across both scan types. For general image quality, fast adaptive 4DCBCT received a mean score of 4.1 ± 1.3, and conventional 4DCBCT received a mean score of 4.3 ± 1.2. The standard deviations for both tumor visibility and general image quality scores were comparable between scan types.

Tumor visibility scores between fast adaptive 4DCBCT and conventional 4DCBCT (p = 0.58) showed no statistically significant differences (p > 0.05). Similarly, general image quality ratings for fast adaptive (p = 0.23) did not show any statistically significant differences from conventional 4DCBCT.

Inter-observer agreement for tumor visibility demonstrated moderate agreement among the six assessors (Fleiss’ κ = 0.31). Image quality scoring showed good reliability across assessors (ICC(2,k) = 0.82), indicating consistent scoring on the 10-point scale.

## 4 Discussion

In lung cancer radiotherapy, 4DCBCT is considered the gold standard for image guidance because it enables the capture of respiratory motion [21]. However, its clinical use is limited by long acquisition times and high radiation doses. To address these limitations, the ADAPT clinical trial aimed to test whether fast adaptive 4DCBCT produces images of similar utility to conventional 4DCBCT [16]. This was achieved using an adaptive acquisition framework that tailors the projection acquisition to each patient's respiratory cycle, combined with motion-compensated reconstruction (MC-MKB). Although motion-compensated reconstruction is not yet widely implemented in routine clinical workflows, the present findings provide clinical evidence supporting its feasibility in a treatment setting. The study hypothesized that fast adaptive 4DCBCT (60–80s, 200 projections) produces equivalent image quality compared to conventional 4DCBCT (4-minute scan time, 1320 projections).

This study compared conventional 4DCBCT with a fast adaptive protocol using data from 30 lung cancer patients across two treatment fractions. Expert reviewers assessed tumor visibility and overall image quality using a structured evaluation framework. The results support the primary hypothesis, demonstrating that fast adaptive 4DCBCT can achieve comparable tumor visibility and image quality to conventional 4DCBCT without a statistically significant difference between protocols among the six assessors (Fleiss’ κ = 0.31).

Tumor visibility ratings did not differ significantly between fast adaptive and conventional 4DCBCT. Although fast adaptive 4DCBCT showed slightly lower overall image quality scores, this difference was not statistically significant within the study sample size. The finding that tumor visibility was rated as limited in 15% of conventional scans suggests that patient-specific factors may influence perceived image quality more strongly than the acquisition protocol itself.

The Sparse-view Reconstruction (SPARE) challenge [22] demonstrated that high-quality 4D imaging from one-minute acquisitions is feasible using advanced reconstruction algorithms. However, many methods relied on prior motion information from planning 4DCT datasets, which may limit robustness when breathing patterns and tumor motion trajectories change between simulation and treatment fractions, as previously reported in thoracic radiotherapy studies [24]. Fast adaptive 4DCBCT differs in that it derives motion information directly from the on-board projection data acquired during treatment, enabling patient specific adaptation without dependence on external motion priors. Similar reductions in acquisition time have been reported by Bryce-Atkinson et al. [23], supporting the feasibility of shortened 4D imaging protocols.

Tumor visibility and perceived image quality were influenced by patient-specific characteristics rather than acquisition protocol alone. In this cohort, body weights ranged from 62–124 kg; 10 patients had Stage I disease, 14 had Stage II/III disease, and 6 had Stage IV or recurrent disease. A larger study stratified by these factors may be required to determine whether image utility is compromised in specific subgroups, such as reduced tumor conspicuity, increased motion artefacts, or limitations in target localization affecting clinical decision-making.

The present study extends findings from the first adaptive 4DCBCT implementation reported by O’Brien et al. [16] by providing an independent clinical evaluation of tumor visibility and image quality. While previous work demonstrated technical feasibility, the current results confirm that these improvements translate into clinically comparable image utility under routine conditions.

Other approaches to shorten acquisition time have relied on increasing gantry rotation speed or reducing projection density, which may introduce motion blurring and artefacts, particularly in patients with irregular respiration [22], [23]. In contrast, fast adaptive 4DCBCT maintains constant gantry velocity while adjusting projection sampling to the patient’s breathing pattern, providing more reliable image quality across respiratory conditions.

This study has several limitations. The cohort included 30 patients from a single institution using one linear accelerator, which may limit generalizability despite reflecting routine clinical practice. Patient-specific factors such as body habitus, tumor stage, and respiratory variability may influence image utility, but subgroup analyses were not performed. Image quality and tumor visibility were assessed qualitatively, introducing subjectivity despite experienced reviewers. Finally, rather than acquiring images on every day of treatment, imaging was performed only on two fractions to enable within-patient comparison of scan protocols without the time and dose increase that would occur with daily imaging in the trial.

Future work may integrate real-time adaptive acquisition with advanced iterative and machine learning reconstruction algorithms. Additionally, patient-specific motion prediction models, as proposed previously [24], could improve tumor localization and reduce clinical variability.

In summary, this study demonstrates the clinical feasibility of fast adaptive 4DCBCT, showing comparable tumor visibility and image quality to conventional 4DCBCT under routine conditions. These findings support integration of adaptive acquisition into clinical workflows, with reduced scan duration and projection count.

## CRediT authorship contribution statement

**Sadia Sana:** Writing – review & editing, Writing – original draft, Visualization, Validation, Software, Methodology, Investigation, Formal analysis, Data curation, Conceptualization. **Shalini K. Vinod:** Writing – review & editing, Validation, Resources, Project administration, Methodology, Investigation, Funding acquisition, Data curation, Conceptualization. **Paul Keall:** Writing – review & editing, Supervision, Resources, Project administration, Methodology, Investigation, Funding acquisition, Data curation, Conceptualization. **Vicky Chin:** Writing – review & editing, Visualization, Validation, Software, Investigation, Data curation, Conceptualization. **Nabeeha Chowdhury:** Writing – review & editing, Software, Methodology, Data curation, Conceptualization. **Jan-Jakob Sonke:** Writing – review & editing, Validation, Software, Project administration, Methodology, Investigation, Data curation, Conceptualization. **Christine Tawfik:** Writing – review & editing, Visualization, Investigation, Data curation. **Isabella Franji:** Writing – review & editing, Visualization, Investigation, Data curation. **Rebecca Bartlett:** Writing – review & editing, Visualization, Investigation, Data curation. **Vinh Luong-Poole:** Writing – review & editing, Visualization, Investigation, Data curation. **Owen Dillon:** Writing – review & editing, Visualization, Validation, Supervision, Software, Resources, Methodology, Investigation, Funding acquisition, Formal analysis, Data curation, Conceptualization. **Ricky T. O’Brien:** Writing – review & editing, Writing – original draft, Visualization, Validation, Supervision, Software, Resources, Project administration, Methodology, Investigation, Funding acquisition, Formal analysis, Data curation, Conceptualization.

## Declaration of competing interest

The authors declare that they have no known competing financial interests or personal relationships that could have appeared to influence the work reported in this paper.
